# Impact of COVID-19 Confinement on Physical Activity and Sedentary Behaviour in Spanish University Students: Role of Gender

**DOI:** 10.3390/ijerph18020369

**Published:** 2021-01-06

**Authors:** Ana Rodríguez-Larrad, Asier Mañas, Idoia Labayen, Marcela González-Gross, Ander Espin, Susana Aznar, José Antonio Serrano-Sánchez, Francisco J. Vera-Garcia, Domingo González-Lamuño, Ignacio Ara, Luis Carrasco-Páez, José Castro-Piñero, Mari Carmen Gómez-Cabrera, Sara Márquez, Josep A. Tur, Narcis Gusi, Pedro J. Benito, Diego Moliner-Urdiales, Jonatan R. Ruiz, Francisco B. Ortega, David Jiménez-Pavón, José Antonio Casajús, Jon Irazusta

**Affiliations:** 1 AgeingOn Research Group, Department of Physiology, University of the Basque Country (UPV/EHU), 48940 Leioa, Spain; ana.rodriguez@ehu.eus (A.R.-L.); ander.espin@ehu.eus (A.E.); 2 GENUD Toledo Research Group, Department of Physical Activity and Sport Sciences, Universidad de Castilla-La Mancha, 45071 Toledo, Spain; Asier.Manas@uclm.es (A.M.); ignacio.ara@uclm.es (I.A.); 3 CIBER of Frailty and Healthy Aging (CIBERFES), 28029 Madrid, Spain; 4 Department of Health Sciences, Institute for Innovation & Sustainable Development in Food Chain (IS-FOOD), Navarra Institute for Health Research (IdisNA), Public University of Navarra, Campus de Arrosadía, 31006 Pamplona, Spain; idoia.labayen@unavarra.es; 5 Department of Health and Human Performance, Universidad Politécnica de Madrid, 28040 Madrid, Spain; marcela.gonzalez.gross@upm.es (M.G.-G.); pedroj.benito@upm.es (P.J.B.); 6 PAFS Research Group, Faculty of Sports Sciences, University of Castilla-La Mancha, 45071 Toledo, Spain; susana.aznar@uclm.es; 7 Department of Physical Education, University of Las Palmas de Gran Canaria, 35017 Las Palmas de Gran Canaria, Spain; jose.serrano@ulpgc.es; 8 Research Institute of Biomedical and Health Sciences (IUIBS), 35016 Las Palmas de Gran Canaria, Spain; 9 Sports Research Centre, Department of Sport Sciences, Miguel Hernández University of Elche, 03202 Elche, Spain; fvera@goumh.umh.es; 10 Department of Medical and Surgical Sciences, University of Cantabria, 39011 Santander, Spain; Domingo.gonzalez-lamuno@unican.es; 11 Department of Physical Education and Sports, University of Seville, 41013 Sevilla, Spain; lcarrasco@us.es; 12 GALENO Research Group, Department of Physical Education, Faculty of Education Sciences, University of Cadiz, 11519 Puerto Real, Spain; jose.castro@uca.es; 13 Biomedical Research and Innovation Institute of Cadiz (INiBICA) Research Unit, 11009 Cádiz, Spain; david.jimenez@uca.es; 14 Freshage Research Group, Department of Physiology, Faculty of Medicine, University of Valencia, 46010 Valencia, Spain; Carmen.Gomez@uv.es; 15 CIBERFES, Fundación Investigación Hospital Clínico Universitario/INCLIVA, 46010 Valencia, Spain; 16 Institute of Biomedicine (IBIOMED), University of León, 24071 León, Spain; sara.marquez@unileon.es; 17 Research Group on Community Nutrition and Oxidative Stress, Fundamental Biology and Health Sciences, University of the Balearic Islands, IDISBA & CIBEROBN, 07122 Palma, Spain; pep.tur@uib.es; 18 Department of Didactics of Musical, Plastic and Body Expression, Faculty of Sport Sciences, University of Extremadura, 10003 Cáceres, Spain; ngusi@unex.es; 19 LIFE Research Group, Department of Education and Specific Didactics, University Jaume I, 12071 Castelló de la Plana, Spain; dmoliner@uji.es; 20 PROFITH “PROmoting FITness and Health through Physical Activity” Research Group, Department of Physical Education and Sports, Faculty of Sport Sciences, University of Granada, 18071 Granada, Spain; ruizj@ugr.es (J.R.R.); ortegaf@ugr.es (F.B.O.); 21 MOVE-IT Research Group, Department of Physical Education, Faculty of Education Sciences, University of Cádiz, 11519 Puerto Real, Spain; 22 Department of Physiatry and Nursing, University of Zaragoza, 50009 Zaragoza, Spain; joseant@unizar.es

**Keywords:** pandemic, international physical activity questionnaire, physical exercise, lockdown

## Abstract

During the COVID-19 pandemic, entire populations were instructed to live in home-confinement to prevent the expansion of the disease. Spain was one of the countries with the strictest conditions, as outdoor physical activity was banned for nearly two months. This study aimed to analyse the changes in physical activity and sedentary behaviours in Spanish university students before and during the confinement by COVID-19 with special focus on gender. We also analysed enjoyment, the tools used and motivation and impediments for doing physical activity. An online questionnaire, which included the International Physical Activity Questionnaire Short Form and certain “ad hoc” questions, was designed. Students were recruited by distributing an invitation through the administrative channels of 16 universities and a total of 13,754 valid surveys were collected. Overall, university students reduced moderate (−29.5%) and vigorous (−18.3%) physical activity during the confinement and increased sedentary time (+52.7%). However, they spent more time on high intensity interval training (HIIT) (+18.2%) and mind-body activities (e.g., yoga) (+80.0%). Adaptation to the confinement, in terms of physical activity, was handled better by women than by men. These results will help design strategies for each gender to promote physical activity and reduce sedentary behaviour during confinement periods.

## 1. Introduction

The COVID-19 pandemic led almost all countries to take extraordinary measures to avoid spreading the disease [1]. Entire populations were instructed to live in home-confinement for a number of weeks to months. Spain was one of the countries with the strictest conditions during the pandemic: leaving home was only allowed for essential needs, such as purchasing food or pharmacological supplies and performing specific professional activities. Any other kind of outdoor activity was banned for nearly 2 months. Even individual outdoor physical activity, which was permitted in many other countries, was prohibited in Spain [2]. These public health measures imposed to prevent the expansion of the disease posed a significant challenge for staying physically active [3]. In addition, during the stay at home, leisure interests might have focused on sedentary behaviours around screen activities [4]. This may represent a concern, as just a few days of inactivity can induce muscle loss, neuromuscular damage, insulin resistance and fat deposition [5]. Moreover, home-confinement may have a psychological and social impact on individuals [6].

Regular physical activity has well-known benefits in health and has demonstrated to be effective for preventing the most prevalent non-communicable pathologies [7] and decreasing mortality risk [8,9]. Regarding communicable diseases, physical activity improves the immune response to infections, which could reduce both the risk of infection by SARS-CoV-2 and the severity of COVID-19 symptoms [10]. In contrast, a sedentary lifestyle has the opposite effect, since this may counteract many of the benefits of physical activity [11,12].

University life usually coincides with the transition between adolescence and adulthood, which is crucial in establishing habits that will be maintained throughout life [13]. Students who are more physically active and with better physical fitness exhibit better health-related quality of life [14], health parameters [14,15] and academic performance [16]. In addition, being physically unfit at a young age could also result in a greater risk of disability thirty years later [17]. The confinement and the closure of sports facilities might have affected physical activity and sedentary behaviour of university students and this could have been further exacerbated by the implementation of online classes.

Patterns of physical activity are not equal by gender. Women, compared to men, spend less time on outdoor activities at different ages. Thus, while assessing children and adolescents, Klinker and colleagues showed that girls performed less outdoor activities and that they also spent less time on moderate to vigorous outdoor physical activity than boys [18]. The same tendency was found in the general population [19] and in older adults [20]. Given these results, it was hypothesized that women would be less affected by the home-confinement and that therefore their levels of physical activity would not change as much as men’s.

Most studies performed during the COVID-19 pandemic observed a global reduction in physical activity in children and adolescents [21] and in the general population [22,23], with the reduction especially pronounced in Spain [24]. However, in Belgium, where individual outdoor physical activity was promoted, although sedentary time increased, more people exercised during the confinement than before [25]. Previous studies mainly assessed quantitative aspects of physical activity (i.e., steps taken, time spent on different intensities) and very few considered qualitative aspects, such as type of activity, conditioning factors and tools used to practise it [25]. Analysing both aspects together will increase the understanding of the impact of the confinement on activity patterns. Moreover, with the exception of data provided by companies that manufacture activity trackers [24], few studies, which were performed in the general population [25], evaluated changes in physical activity in large samples, neither did they consider gender to analyse the results. Furthermore, the assessment of physical activity is even more relevant in countries with strict prohibitions to do physical leisure activities outdoors, such as Spain.

University students belong to a group of paramount importance in the social and economic development of society because they will be qualified professionals in the near future. In consequence, habits that could affect their health and wellbeing in the short- and the long-term deserve to be studied. However, few studies, that did not take into account either gender or qualitative aspects [26] have analysed the impact of Covid-19 pandemic on physical activity in this specific population.

The aim of this study was to analyse the changes in patterns of physical activity and sedentary behaviours in Spanish university students before and during the confinement with special focus on gender. As a secondary aim, we analysed the enjoyment, the tools used and motivation and impediments to do physical activity.

## 2. Materials and Methods

This manuscript presents data from a comparative study (ClinicalTrials.gov, NCT04361019), analysing the differences between physical activity-related parameters before and during the confinement due to COVID-19. Data were collected via an online survey [27] between 16 April and 2 May. In this period, the Spanish population was strictly home-confined and only allowed to leave home for essential needs, such as food shopping or purchasing pharmacological supplies, or to perform specific professional activities [2]. Police controlled restrictions about leaving home.

### 2.1. Survey Development and Promotion

The survey was based on the International Physical Activity Questionnaire (IPAQ) Short Form. The survey also included questions regarding qualitative aspects of physical activity and sedentary behaviours of participants before and during the confinement. Students registered at Spanish Universities and living in Spain during the confinement were eligible to participate in the study. University students older than 55 years were excluded from the analysis. Participants were recruited by distributing an invitation through administrative channels of 16 universities and snowball sampling through social media (Twitter, Facebook, Instagram and so on). All participants gave their informed consent before they participated in the study. The study was conducted in accordance with the declaration of Helsinki and the protocol was approved by the Ethics Committee for Human Beings of the University of the Basque Country (M10_2020_078).

### 2.2. Survey Dimension

#### 2.2.1. Sociodemographic, Academic and Anthropometric Data

Data concerning gender, age, height, weight, university, academic degree (Bachelor’s, Master’s, PhD) and the branch of knowledge of the studies were collected.

#### 2.2.2. Physical Activity and Sedentary Behaviour

The time spent on moderate and vigorous physical activities, as well as walking and sedentary time, were assessed using the IPAQ Short Form, which has been validated among Spanish university students [28]. Additionally, time per week in each type of physical activity performed (i.e., aerobic exercises, strength training, high intensity interval training (HIIT, a type of training involving repeated bouts of high intensity effort followed by varied recovery times), sports or mind-body exercises such as Pilates, Tai-Chi and Yoga) and time per day spent on leisure- and study-related screen activities were collected. For these variables, each of the questions had to be responded twice: firstly, referring to “during the confinement” and consecutively to “before the confinement” periods.

The following qualitative information about physical activity during the confinement was also recorded: (a) perceived intensity and enjoyment of physical activity (close-ended questions with three options: “lower than”, “higher than” or “equal to” before the confinement), (b) tools used for physical activity (close-ended questions with yes/no response options such as “equipment for aerobic exercise”, “equipment for strength exercise”, “active videogames”, “computer applications”, “TV programs” or “social networks” and so on), (c) reasons for doing physical activity (close-ended questions with yes/no response options such as “it is important for my health”, “it is important to my image”, “it helps me against stress and anxiety”, “I spend more time sitting”, “I have more time to exercise” and so on) and (d) reasons for not doing physical activity (close-ended questions with yes/no response options such as “it is not a priority for me, “I cannot go out and do my usual exercise”, “I do not know how to exercise at home”, “I have no material resources”, “I do not have enough space” and so on). These questions were designed ad hoc, considering the specific confinement situation.

### 2.3. Statistical Analysis

Continuous variables are shown using mean (+standard deviation). Normality of the distribution of values was checked by the Kolmogorov–Smirnoff test. For statistical analysis, non-normal data were square root-transformed. A paired t-test was used to compare continuous parameters before and during the confinement in the entire sample. In this test, effect size was calculated by Cohen’s d. Values for Cohen’s d of 0.2, 0.5 and 0.8 were considered small, medium and large, respectively [29]. The interaction of gender in the changes before and during the confinement were analysed by a mixed design (gender × time) ANCOVA, including the branch of knowledge of the studies as a covariable. As a result, η^2^ was calculated to analyse the effect size. Values for η^2^ of 0.01, 0.06, 0.13 were considered small, medium and large, respectively [30]. Categorical data were expressed by percentages in each category, while values between men and women were compared by the χ^2^ test and effect size by φ or Crammer’s V. In these tests, values of 0.1, 0.3 and 0.5 were considered small, medium and large, respectively [31]. For all analyses, significance level was set at *p* < 0.05. Statistical analysis was performed using IBM SPSS Statistics for Windows version 24.0 (IBM Corp., Armonk, NY, USA).

## 3. Results

### 3.1. Descriptive Analysis of Participants

Table 1 shows the descriptive data of the sample. After sending an email to almost 500,000 students of the 16 universities that participated in the study, a total of 13,754 valid surveys were collected. The average age of respondents was 22.6 years for women (range: 18–54) and 23.2 years for men (range: 18–54): 65.2% of participants were women, 34.3% were men and 0.5% did not declare gender.

### 3.2. Changes in the Whole Sample

Table 2 shows the changes in the time spent on each intensity and type of activity, before and during the confinement. University students spent less time on moderate (−29.5%; Cohen’s d = 0.210) and vigorous (−18.3%; Cohen’s d = 0.113) physical activity during the confinement than before it. A more drastic (−84.3%; Cohen’s d = 1.340) reduction in walking time was also observed. Regarding type of physical activity, whereas the time spent on aerobic activity (−31.3%; Cohen’s d = 0.277) and sports (−87.4%; Cohen’s d = 0.582) was lower during the confinement, university students performed more HIIT (+18.2%; Cohen’s d = 0.141) and mind-body activities (+80.0%, Cohen’s d = 0.362), while maintaining strength exercises (−0.7%) in this period. Sedentary time increased (+52.7% Cohen’s d = 0.998) during the confinement, including leisure-time (+71.9%; Cohen’s d = 1.244) and study-related (+37.1%; Cohen’s d = 0.587) screen time.

Table 3 shows the qualitative information about physical activity during the confinement. Participants who reported that the intensity of aerobic (51.3%) and strength (38.8%) exercise was lower during the confinement than before outnumber those who reported the opposite (29.8% and 35.2%, respectively). However, regarding HIIT, the proportion of participants who felt that the intensity was higher (30.5%) or lower (30.2%) during the confinement was similar. In addition, while 45.5% of the participants enjoyed doing physical activity less during the confinement than before it, only 27.1% enjoyed it more. The main reasons for doing physical activity were to promote health (74.4%) and to reduce stress (65.5%), while the main reasons for not engaging in physical activity were legal restrictions on outdoor activities (9.3%) and lack of time (7.6%). Finally, tutorials on social media (63.9%) and equipment used for strength exercises (52.3%) were the most frequently used tools.

### 3.3. Gender-Related Differences

Table 4 shows changes in the time spent on different intensities and types of physical activity and sedentary behaviour before and during the confinement in men and women. The time spent on moderate (η^2^ for time × group = 0.009) and vigorous (η^2^ for time × group = 0.02) physical activities decreased more in men (−39.2% and −31.8%, respectively) than in women (−24.6% and −9.1%, respectively). However, the reduction in walking time was slightly but significantly larger in women than in men (−84.8% vs. −83.2%; η^2^ for time × group = 0.003). In addition, while men decreased the time spent on strength exercise (−13.3%) and they maintained HIIT activities, women increased the time spent on strength exercise (+10.1%) and HIIT (+32.2%) during the confinement (η^2^ for time × group = 0.011 for strength and η^2^ for time × group = 0.007 for HIIT). Similarly, men decreased the time spent on aerobic activities more (−48.6%) than women (−19.9%; η^2^ for time × group = 0.03). Men (−89.7% vs. −85.3%) decreased the time doing sports more than women did (η^2^ for time × group = 0.057). The time doing mind-body activities increased more in women (+93.3%) than in men (+43.3%) (η^2^ for time × group = 0.021). Finally, sedentary time (η^2^ for time × group = 0.002) and leisure screen time (η^2^ for time × group = 0.003) increased slightly more in men (+54.7% and +76.7%) than in women (+51.5% and +69.4%).

Table 5 shows the differences between women and men regarding the qualitative information about physical activity during the confinement. More women than men (Krammer’s V = 0.2) reported that they increased the intensity of aerobic (36.4% vs. 17.5%), strength (37.2% vs. 31.5%) and HIIT (33.6% vs. 24.2%) activities during the confinement (Table 5); there were also more women (33.6%) than men (15.0%) who increased their enjoyment of physical activity during this period (Krammer’s V = 0.221). Social media were more widely used for practising physical activity in women (76.6%; φ = 0.362), with equipment for strength exercises being more common in men (58.7%; φ = 0.092). Health promotion and reduction of stress were the most frequent reasons for doing physical activity in both genders, with the first being more frequent in men (75.9%; φ = 0.024) and the second in women (67.5%; φ = 0.083). Finally, lack of time for women (7.7%; φ = 0.027) and restrictions on going out for men (13.3%; φ = 0.030) were the most common problems in terms of not doing physical activity.

## 4. Discussion

The present study shows that university students slightly decreased the time spent on moderate and vigorous physical activities, but they considerably decreased the time spent on walking and doing sports during the confinement. Moreover, students substantially increased sedentary time and leisure-time screen activities during this period. In contrast, they increased the time doing HIIT and mind-body activities and they maintained strength exercise. Women adapted their pattern of physical activity to the confinement better; they reduced the time spent on moderate and vigorous physical activity less and they increased the time doing strength exercise, HIIT and mind-body activities more than men did. In addition, more women than men enjoyed doing physical activity more during than before the pandemic. We consider that these results should be taken into account to promote physical activity in putative future scenarios where strict confinements are needed.

### 4.1. Changes in the Whole Sample

The majority of previous studies showed decreases in physical activity during the confinement caused by the COVID-19 pandemic. In this regard, an international online survey, carried out in countries with different measures regarding outdoor physical activity, demonstrated that the confinement reduced moderate and vigorous physical activity around 35% in adults [22]. Similarly, more than a half of the people in a French study decreased physical activity during the confinement [32]. However, increases in the levels of physical exercise were reported among Belgian adults during the confinement [25]. It is noticeable that in this latter mentioned country the government promoted outdoor physical activity. Considering only studies carried out in countries where physical exercise was forbidden, our results contrast with a study carried out in Chinese children and adolescents that found greater reduction in moderate and vigorous physical activity during the pandemic [21] than in our research. However, the decrease found in the present study was similar to that found by Galle and co-workers in a sample of Italian undergraduate students [26]. In this regard, it was demonstrated that people with a higher level of education are more aware of the benefits of physical activity [33].

The increase in time spent on HIIT found in the present study may be one reason why there was only a slight decrease in vigorous physical activity during the confinement. Considering that sports centres, places where HIIT is more frequently practised, were closed during the pandemic, this increase seems particularly striking. However, the wide use of social media (i.e., YouTube tutorials) could have encouraged engagement in physical activity and particularly in HIIT, during the confinement. In this regard, a very recent editorial reported that the interest for the terms “exercise” and “HIIT” in Google searches peaked during the first 2 weeks of the confinement, with both reaching their highest Google Trends record since 2004 [34]. These results also agree with the fact that HIIT was considered as the second fitness trend for physical activity in 2020 according to the American College of Sports Medicine [35]. The success of social media in maintaining physical activity among the population during the confinement suggests that there is a need for developing and ensuring the quality of physical activity proposals in this format. In addition, online proposals should also focus on other collectives, such as elderly people, who also need simple and safe ways to stay physically active at home [36,37].

There was a substantial increase in the sedentary time and a decrease in the time walking. Considering the conditions of the confinement in Spain, it is understandable that the reduction in walking was greater than in other studies [22,23]. However, the decrease found in the present study is worrying as it was even more pronounced than that found for Fitbit users in Spain [24]. Considering that the data from the Fitbit study encompassed the general population, it seems that university students have been a particularly affected population, so it could be thought that the implementation of online classes may have negatively affected walking and sedentary time. Moreover, the time spent on screen activities increased drastically in this study, especially activities related to leisure-time. These results may arouse concern because some of the leisure-time screen activities could be potentially addictive for university students [38] and this could increase their sedentary behaviour in the future. It is known that, regardless of physical activity, sedentary time is associated with worse health outcomes [11,12]. This could imply that the potential health benefits of doing physical activity during the confinement could have been attenuated by increased sedentary time. In this regard, avoiding sitting for long periods of time and taking brief movement or activity breaks during the day have been proposed as effective strategies to reduce sedentary behaviour [3].

### 4.2. Gender-Related Differences

We found more gender-related differences from the effects of the confinement. Overall, women seem to have adapted their physical activity better during the confinement. The differences in the changes between men and women were small, but significant and consistent in the great majority of analysed parameters. The same gender-specific tendency during the COVID-19 pandemic was found between male and female adolescents in Croatia [39]. As stated, our data show that women enjoyed physical activity during the confinement more than men did and that they used social media for physical activity more often. These gender-related differences with social media seem to be specific for physical activity: social media in Spain for young people is well balanced between men and women [40]. In addition, women reported that one reason for doing physical activity is that they found new resources. In comparison, the reasons given by men for not practising physical activity included not being able to go outdoors. In this regard, it is noticeable that men usually engage in more outdoor activities than women at different ages [18,19,20]. During the confinement, women’s lower dependency on outdoor environments might have encouraged them to reduce physical activity less than men. Considering these results, strategies to promote physical activity during and after confinements should be adapted to each gender.

### 4.3. Strengths and Limitations

The main strength of this research is that the study collects the most relevant questions on the frequency and intensity of physical activity from previously validated questionnaires (i.e., IPAQ), but also qualitative and descriptive data for both active and sedentary lifestyles. In addition, the population under study, i.e., a large sample of university students, is a group of people of paramount importance for the socioeconomic future of our society. Finally, comparing with other published studies about the topic, the number of participants was higher and results were analysed taking into account gender.

Certain limitations must be acknowledged. These results cannot be directly extrapolated to other populations, as parameters such as age, educational level and the use of internet resources are not comparable. Furthermore, it must be taken into account that restrictions during the confinement in Spain were not the same as in other countries. In consequence, they cannot be directly extrapolated to other countries. In addition, physical activity was self-reported and the university students who responded might be those who were more active. Both of these points may have led to an overestimation of the physical activity actually carried out [41]. However, the sample size and the timing of the collected information did not allow us to use objective methods, as a function of both logistics and time.

## 5. Conclusions

University students decreased time spent on moderate and vigorous physical activity during the COVID-19 confinement. In contrast, they increased HIIT and mind-body activities, maintained strength exercise and widely used social media, as a support for doing exercise. Taking into account that HIIT is commonly offered on social media, our results suggest that the diffusion of this type of physical exercise via online channels could help to maintain people physically active, while they need or prefer to stay at home. 

On the other hand, university students increased sedentary and leisure screen times during the confinement. Due to the direct impact of sedentary behaviour on health [11,12], its reduction should be strongly promoted in the context of confinements. Moreover, given the addictive nature of some screen activities [38], their increase during the confinement could lead to an increase in sedentary behaviours in the long-term. 

Finally, women adapted to the confinement better than men did; they reduced the time spent on physical activity less, they enjoyed doing physical activity more and they used social media for doing physical activity more frequently as well. As a consequence, strategies to promote physical activity during confinement periods should be tailored according to the gender.

## Figures and Tables

**Table 1:** Descriptive data of study sample.

Variable	Overall	Women	Men
Age (years), mean (SD) *	*n* = 13,754	*n* = 8960	*n* = 4728
	22.8 (5.3)	22.6 (4.9)	23.2 (5.8)
Body Mass Index (kg/m^2^), mean (SD) *	*n* = 13,623	*n* = 8859	*n* = 4699
	22.6 (3.3)	22.0 (3.3)	23.5 (3.1)
Academic degree, *n* (%) *	*n* = 13,753	*n* = 8959	*n* = 4728
Bachelor’s degree	11,360 (82.6)	7484 (83.5)	3819 (80.8)
Master’s degree	1322 (9.6)	803 (9.0)	513 (10.9)
PhD	995 (7.2)	613 (6.8)	379 (8.0)
Other	76 (0.6)	59 (0.7)	17 (0.4)
Branch of knowledge, *n* (%) *	*n* = 13,629	*n* = 8860	*n* = 4704
Arts and Humanities	1315 (9.6)	1055 (11.9)	243 (5.2)
Engineering and Architecture	3238 (23.8)	1334 (15.1)	1890 (40.2)
Experimental sciences	1346 (9.9)	851 (9.6)	489 (10.4)
Health sciences	3612 (26.5)	2869 (32.4)	730 (15.5)
Social and Legal sciences	3303 (24.2)	2441 (27.6)	847 (18.0)
Physical Activity and Sports sciences	815 (6.0)	310 (3.5)	505 (10.7)
Housing type, *n* (%) *	*n* = 13,577	*n* = 8848	*n* = 4645
Apartment in multi-storey building	8819 (65.1)	5823 (65.8)	2946 (63.4)
Semi-detached house	2480 (18.3)	1575 (17.8)	896 (19.3)
Isolated family house	2258 (16.7)	1450 (16.4)	803 (17.3)
Coexistence at home, *n* (%) *	*n* = 13,741	*n* = 8951	*n* = 4724
Parents	3663 (26.7)	2366 (26.4)	1277 (27.0)
Parents and siblings	6348 (46.2)	4141 (46.3)	2180 (46.1)
Partner	935 (6.8)	692 (7.7)	235 (5.0)
Roommates	1501 (10.9)	957 (10.7)	537 (11.4)
Alone	458 (3.3)	261 (2.9)	196 (4.1)
Other	836 (6.1)	534 (6.0)	299 (6.3)
Coexistence in unit size, *n* (%) *	*n* = 13,703	*n* = 8933	*n* = 4705
1 person	429 (3.1)	229 (2.6)	197 (4.2)
2 people	1904 (13.9)	1331 (14.9)	562 (11.9)
3 people	3878 (28.3)	2513 (28.1)	1346 (28.6)
4 people	5557 (40.6)	3631 (40.6)	1904 (40.5)
≥5 people	1935 (14.1)	1229 (13.8)	696 (14.8)
COVID-19 related information, *n* (%)			
Self-reported symptoms	*n* = 13,734	*n* = 8947	*n* = 4721
	846 (6.2)	535 (6.0)	302 (6.4)
Diagnosed by health professional	*n* = 13,718	*n* = 8936	*n* = 4716
	95 (0.7)	61 (0.7)	32 (0.7)
Coexistence at home with affected	*n* = 13,741	*n* = 8953	*n* = 4722
	427 (3.1)	264 (2.9)	161 (3.4)

Note: * *p* < 0.05, statistically significant difference (Student’s T or χ^2^ tests) between women and men.

**Table 2:** Participants’ reported Physical Activity, Exercise and Sedentary Time during COVID-19 confinement. Overall sample.

Variable	Before the Confinement	During the Confinement	Change	Student’s Paired T Test (*p*)	Cohen’s d
IPAQ-SF					
Vigorous PA (min/week)	327 (374)	267 (309)	−18.3%	<0.001	0.113
Moderate PA (min/week)	376 (563)	265 (408)	−29.5%	<0.001	0.210
Walking time (min/week)	766 (820)	120 (318)	−84.3%	<0.001	1.340
Sedentary time (min/day)	357 (178)	545 (200)	+52.7%	<0.001	0.998
Exercise					
Aerobic (min/week)	208 (240)	143 (179)	−31.3%	<0.001	0.277
Strength (min/week)	136 (189)	135 (171)	−0.1%	<0.001	0.048
HIIT (min/week)	66 (125)	78 (145)	+18.2%	<0.001	0.142
Mind-body (min/week)	40 (105)	72 (143)	+80.0%	<0.001	0.361
Sports (min/week)	95 (193)	12 (68)	−87.4%	<0.001	0.582
Screen time					
Leisure (min/day)	217 (140)	373 (202)	+71.9%	<0.001	1.244
Study, work (min/day)	251 (149)	344 (165)	+37.1%	<0.001	0.587

Notes: Data are presented as mean (SD). Sample size ranges between 12,526 and 13,491 in the different variables. HIIT, High Intensity Interval Training; IPAQ-SF, International Physical Activity Questionnaire Short Form; min, minutes; PA, Physical Activity.

**Table 3:** Participants’ perceptions and resources regarding the practice of physical exercise during the COVID-19 confinement. Overall sample.

Variable	*n* (%)
Intensity, aerobic (*n* = 13,183)	
Same as before	2493 (18.9)
Higher than before	3929 (29.8)
Lower than before	6761 (51.3)
Intensity, strength (*n* = 13,412)	
Same as before	3480 (25.9)
Higher than before	4722 (35.2)
Lower than before	5210 (38.8)
Intensity, HIIT (*n* = 12,819)	
Same as before	5046 (39.4)
Higher than before	3904 (30.5)
Lower than before	3869 (30.2)
Enjoyment when exercising (*n* = 13,367)	
Same as before	3660 (27.4)
Higher than before	3627 (27.1)
Lower than before	6080 (45.5)
Available spaces for exercising (*n* = 13,754)	
Room, hallways	8833 (64.2)
Living room	6523 (47.4)
Courtyard	1043 (7.6)
Garden/Exterior free space	2928 (21.3)
Other	1696 (12.3)
Tools used for exercising (*n* = 13,754)	
Equipment for aerobic exercise	3617 (26.3)
Equipment for strength exercises	7198 (52.3)
Active videogames	1001 (7.3)
Computer applications	2776 (20.2)
TV programs	724 (5.3)
Social networks	8790 (63.9)
Reasons to exercise (*n* = 13,754)	
It is important for my health	10,231 (74.4)
It is important to my image	5769 (41.9)
It helps me against stress and anxiety	8876 (64.5)
I spend more time sitting	4305 (31.3)
I have more time to exercise	4768 (34.7)
I have found different resources	3013 (21.9)
My food intake is higher	2447 (17.8)
My environment pushes me to it	1252 (9.1)
Reasons not to exercise (*n* = 13,754)	
It is not a priority for me	639 (4.6)
I cannot go out and do my usual exercise	1282 (9.3)
I do not know how to exercise at home	368 (2.7)
I have no material resources	768 (5.6)
I do not have enough space	986 (7.2)
I have less time to exercise	1050 (7.6)
My health state prevents me	209 (1.5)

Note: HIIT, High Intensity Interval Training.

**Table 4:** Participants’ reported physical activity, exercise and sedentary time, during the COVID-19 confinement. Comparison between women and men.

Variable	Women			Men			ANCOVA g × t (*p*) †	η2
	B.C.	D.C.	Change	B.C.	D.C.	Change		
IPAQ-SF								
Vigorous PA (m/w) ‡	296 (372)	269 (319)	−9.1%	386 (371)	263 (290) ¶	−31.9%	<0.001	0.020
Moderate PA (m/w) ‡§	385 (575)	290 (420) ¶	−24.7%	359 (538)	218 (382) ¶	−39.3%	<0.001	0.009
Walking time (m/w) ‡§	803 (845)	122 (310) ¶	−84.8%	697 (764)	117 (332) ¶	−83.2%	<0.001	0.003
Sedentary time (m/d) ‡§	353 (175)	535 (197) ¶	+51.6%	364 (184)	563 (205) ¶	+54.7%	<0.001	0.002
Exercise								
Aerobic (m/w) ‡§	196 (229)	157 (179) ¶	−19.9%	230 (256)	119 (177) ¶	−48.3%	<0.001	0.032
Strength (m/w) ‡§	109 (174)	120 (161) ¶	+10.1%	187 (205)	162 (185) ¶	−13.4%	<0.001	0.011
HIIT (m/w) ‡§	62 (122)	82 (151) ¶	+32.3%	72 (131)	72 (132)	±0.0%	<0.001	0.007
Mind-body (m/w) ‡§	45 (114)	87 (155) ¶	+93.3%	30 (84)	43 (111) ¶	+43.3%	<0.001	0.021
Sports (m/w) ‡§	68 (169)	10 (63) ¶	−85.3%	145 (224)	15 (76) ¶	−89.7%	<0.001	0.057
Screen time								
Leisure (m/d) §	216 (141)	366 (200) ¶	+69.4%	219 (138)	387 (204)	+76.7%	<0.001	0.003
Study, work (m/d) ‡§	256 (149)	349 (165) ¶	+36.3%	243 (149)	334 (165) ¶	+37.4%	0.369	<0.001

Notes: Data are presented as mean (SD). Sample size ranges between 8148 and 8811 in women and 4356 and 4680 in men in the different variables. B.C., before the confinement; D.C., during the confinement; g x t, Group per Time interaction; HIIT, High Intensity Interval Training; IPAQ-SF, International Physical Activity Questionnaire Short Form; m/d, minutes/day; m/w, minutes/week; PA, Physical Activity. † Adjusted for branch of knowledge. ‡ *p*<0.05 (Student’s independent T test) between women and men in before the confinement. § *p* < 0.05 (Student’s independent T test) between women and men in during the confinement. ¶ *p* < 0.05 (Student’s paired T test) between before and during confinement.

**Table 5:** Participants’ perceptions and resources regarding the practice of physical exercise during the COVID-19 confinement. Comparison between women and men.

Variable	Women	Men	χ^2^ (*p*)	Phi/Cramer’s V
Intensity, aerobic	*n* = 8610	*n* = 4510		
Same as before	1561 (18.1)	917 (20.3)	<0.001	0.200
Higher than before	3132 (36.4)	787 (17.5)		
Lower than before	3917 (45.5)	2806 (62.2)		
Intensity, strength	*n* = 8735	*n* = 4612		
Same as before	2459 (28.2)	1000 (21.7)	<0.001	0.120
Higher than before	3251 (37.2)	1455 (31.5)		
Lower than before	3025 (34.6)	2157 (46.8)		
Intensity, HIIT	*n* = 8365	*n* = 4393		
Same as before	3288 (39.3)	1730 (39.4)	<0.001	0.108
Higher than before	2809 (33.6)	1084 (24.7)		
Lower than before	2268 (27.1)	1579 (35.9)		
Enjoyment when exercising	*n* = 8704	*n* = 4597		
Same as before	2422 (27.8)	1219 (26.5)	<0.001	0.221
Higher than before	2925 (33.6)	690 (15.0)		
Lower than before	3357 (38.6)	2688 (58.5)		
Available spaces for exercising	*n* = 8960	*n* = 4728		
Room, hallways	5778 (64.5)	3009 (63.6)	0.327	0.008
Living room	4684 (52.3)	1812 (38.3)	<0.001	0.133
Courtyard	615 (6.9)	425 (9.0)	<0.001	0.038
Garden, exterior free space	1955 (21.8)	967 (20.5)	0.064	0.016
Other	980 (10.9)	709 (15.0)	<0.001	0.059
Tools used for exercising	*n* = 8960	*n* = 4728		
Equipment for aerobic exercise	2314 (25.8)	1295 (27.4)	0.048	0.017
Equipment for strength exercises	4396 (49.1)	2777 (58.7)	<0.001	0.092
Active videogames	691 (7.7)	304 (6.4)	0.006	0.023
Computer applications	1912 (21.3)	853 (18.0)	<0.001	0.039
TV programs	584 (6.5)	135 (2.9)	<0.001	0.078
Social networks	6860 (76.6)	1893 (40.0)	<0.001	0.362
Reasons to exercise	*n* = 8960	*n* = 4728		
It is important for my health	6603 (73.7)	3590 (75.9)	0.004	0.024
It is important to my image	3748 (41.8)	2002 (42.3)	0.563	0.005
It helps me against stress and anxiety	6044 (67.5)	2795 (59.1)	<0.001	0.083
I spend more time sitting	3071 (34.3)	1212 (25.6)	<0.001	0.089
I have more time to exercise	3466 (38.7)	1284 (27.2)	<0.001	0.115
I have found different resources	2438 (27.2)	559 (11.8)	<0.001	0.177
My food intake is higher	1787 (19.9)	652 (13.8)	<0.001	0.076
My environment pushes me to it	859 (9.6)	390 (8.2)	0.010	0.022
Reasons not to exercise	*n* = 8960	*n* = 4728		
It is not a priority for me	378 (4.2)	256 (5.4)	0.002	0.037
I cannot go out and do my usual exercise	643 (7.2)	627 (13.3)	<0.001	0.027
I do not know how to exercise at home	223 (2.5)	143 (3.0)	0.065	0.100
I have no material resources	387 (4.3)	374 (7.9)	<0.001	0.016
I do not have enough space	589 (6.6)	387 (8.2)	<0.001	0.075
I have less time to exercise	688 (7.7)	349 (7.4)	0.532	0.030
My state of health prevents me	145 (1.6)	61 (1.3)	0.134	0.005

Notes: Data are presented as *n* (%). HIIT, High Intensity Interval Training.
